# Future Directions in Diagnosis, Prognosis and Disease Monitoring of Adrenocortical Carcinoma: Novel Non-Invasive Biomarkers

**DOI:** 10.3389/fendo.2021.811293

**Published:** 2022-02-01

**Authors:** Yuling Cheng, Wei Kou, Dandan Zhu, Xinbo Yu, Yu Zhu

**Affiliations:** Department of Urology, Ruijin Hospital, Shanghai Jiao Tong University School of Medicine, Shanghai, China

**Keywords:** adrenocortical carcinoma, non-invasive biomarker, microRNA, steroid profiling, diagnosis, prognosis, disease monitoring

## Abstract

Adrenocortical carcinoma (ACC) is a rare endocrine malignancy with frequent metastatic spread and poor prognosis. The disease can occur at any age with unexpected biological behavior. Recent genome-wide studies of ACC have contributed to our understanding of the disease, but diagnosis of ACC remains a challenge, even for multidisciplinary expert teams. Patients with ACC are frequently diagnosed in advanced stages and have limited therapeutic options. Therefore, for earlier diagnosis and better clinical management of adrenocortical carcinoma, specific, sensitive, and minimal invasive markers are urgently needed. Over several decades, great efforts have been made in discovering novel and reliable diagnostic and prognostic biomarkers including microRNAs, steroid profilings, circulating tumor cells, circulating tumor DNAs and radiomics. In this review, we will summarize these novel noninvasive biomarkers and analyze their values for diagnosis, predicting prognosis, and disease monitoring. Current problems and possible future application of these non-invasive biomarkers will also be discussed.

## Introduction

Adrenal tumors are common diseases, affecting 3~10% human population (1–3). The majority of adrenal tumors are small benign nonfunctional adrenocortical adenomas, and a small fraction of adrenal tumors are adrenocortical carcinomas (1, 2). Adrenocortical carcinoma is a rare malignant tumor with an annual incidence of 0.7-2 cases per million individuals (4–6). It occurs at any age, with two peak incidences: the first one in childhood, mainly derived from hereditary syndromes such as the Li-Fraumeni and Beckwith-Wiedemann syndrome, and the second one between 40 and 60 years of age (3, 4, 7). Women are more frequently affected (55%-60%) than men (8, 9).

ACCs often show aggressive biological behavior, and 50%-70% of patients with ACC have symptoms and signs of hormone excess. Two thirds of them have glucocorticoids and or androgens overproductions (3, 4, 8). A small fraction of these patients have pure androgen excess while estrogen or mineralocorticoid excess are very rare (3). Symptoms of ACC patients who have hormone overproduction usually present with an abdominal mass, weight loss, and other constitutional symptoms (1, 10). One thirds of patients presents with nonspecific symptoms that are related to local tumor growth or spread of tumor to surrounding or distant tissues (2). The German ACC Registry has demonstrated that the median age of primary diagnosis of adrenocortical carcinoma is 46.7 years (10). The conventional diagnosis of adrenocortical carcinoma often relies on imaging and hormonal features and has significant limitations. Owing to the development of imaging techniques, more and more adrenal incidentalomas have been discovered. After exclusion of nonfunctioning benign adrenocortical adenoma, a multidisciplinary discussion is warranted. A multidisciplinary team is able to provide all physicians required for diagnosis and treatment of ACC, which could provide a close and personalized management for patients with ACC (11). Early diagnosis associated with surgical tumor removal has been proven as the best option for ACC treatment (12–14).

Genetic analysis and studies have made great advances in the understanding of the disease. However, the patients with ACC are still diagnosed in advanced stage and have poor prognosis with a 5-year mortality rate of approximately 75% to 90% (15–17). Therefore, for better management of ACC, it is mandatory to discover sensitive, specific, and noninvasive biomarkers that can contribute to early diagnosis of ACC. The best biomarkers should not only be able to distinguish malignancy from benign lesions, but also to provide prognostic information and monitor the disease. The aim of this review was to summarize non-invasive biomarkers of adrenocortical cancer, including microRNAs, steroid metabolomics, circulating tumor cells, circulating tumor DNAs, and radiomics. Meanwhile, current problems and possible future applications will also be discussed.

## MicroRNAs

### Biogenesis and Function of miRNAs

**Figure 1:**
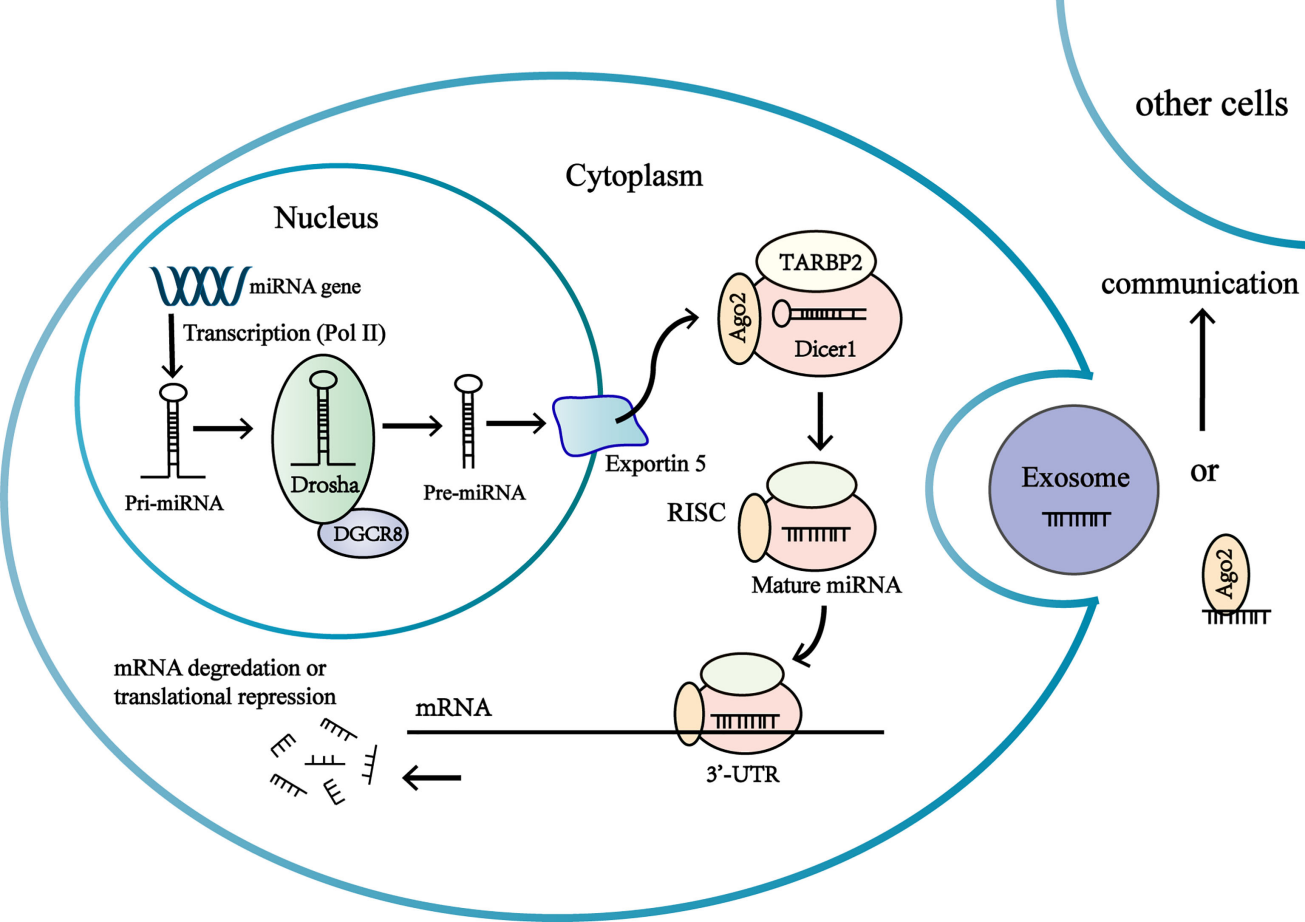
Biogenesis and function of miRNAs in the adrenocortical tumor cells. MiRNA genes are transcribed as primary miRNA (pri-miRNA) in nucleus. Pri-miRNA is processed by multiprotein complex to produce a precursor miRNA (pre-miRNA). The multiprotein complex consists of a double-stranded RNA (dsRNA)-binding protein DGCR8, a nuclear RNase III enzyme Drosha. The pre-miRNAs are transported from nucleus to the cytoplasm by exportin-5 (Exp-5). In the cytoplasm, the pre-miRNA are processed by the RNase III endonuclease Dicer protein and the double-stranded transactivation-responsive RNA-binding protein (TRBP), producing a short double-stranded (ds) miRNA duplex. One strand of miRNA duplex is next loaded into an Ago protein to form RISC, while the other one is degraded by cellular nuclease. RISC complex consists of Dicer, Argonaute 2 (Ago2), and the dsRNA-binding protein TRBP. The association of the miRNA-RISC complex binding to 3’-untranslated region (3’-UTR) of target mRNA leads to inhibition of protein translation or degradation of the mRNA. Extracellular miRNAs in extracellular vesicles or associated with RNA-binding proteins are involved in intercellular communications.

MicroRNAs (miRNA) are small non-coding(~19-24 nucleotides) RNAs (18), most of which undergo a sophisticated maturation process and interact with the 3’untranslated region(3’-UTR) of target messenger RNAs to regulate gene expression at posttranscriptional levels (19, 20). MiRNAs regulate gene expression by inhibiting translation of mRNAs or inducing the degradation of mRNAs (19) (**Figure 1**). Interaction of miRNAs with other regions of mRNA (the 5’ UTR, coding sequence, gene promoters) has also been discovered (19). The same miRNAs can bind with different mRNAs, and miRNAs often act in a synergistic manner to regulate genes expression (21). Expressions of miRNA are tissue specific and the same miRNA can function as oncogenic or tumor suppressor in different tissues (22). One third coding genes are regulated by miRNAs, which means miRNAs take part in a majority of biological processes including proliferation, differentiation and apoptosis (20, 23). Changes in miRNA expression may be associated with tumor development and progression in adrenocortical carcinoma (16, 24).

Over the past decade, novel studies have found that miRNAs can be extracted from various body fluids (blood, urine, feces, breast milk, saliva, tears, etc.) and can be detected with minimal invasion (23, 25–27). Observations have revealed that microRNAs enter body fluids *via* passive release (inflammation or necrosis) or *via* active secretion. Actively secreted miRNAs are either packed in extracellular membrane vesicles (EV) (microvesicles, exosomes, apoptotic bodies) or attached into macromolecular complexes like Argonaute 2 (AGO2) protein and high density lipoprotein (HDL) (27–29) (**Figure 1**). Extracellular miRNAs in extracellular vesicles or associated with high density lipoprotein have been discovered to be transferred to another cell and regulate gene expressions, thus circulating miRNAs might act as epigenetic ‘hormones’ by influencing gene expressions, even in distant cell or tissues (21, 29, 30) (**Figure 1**). However, the mechanisms of regulating miRNA to be secreted into body fluids are poorly understood (21).

### Circulating MiRNAs

To find miRNAs biomarkers for diagnosis of ACC, several studies have investigated the different expressions of tissue miRNAs in benign and malignant adrenocortical tumors. Among these studies, miR-483-5p, miR-483-3p, miR-210, and miR-503 are most consistently overexpressed in ACC, while miR-195 are found to be underexpressed in ACC (24, 31–38). The results have suggested that miRNAs may be a promising biomarkers for diagnosis of adrenocortical carcinoma.

**Figure 2:**
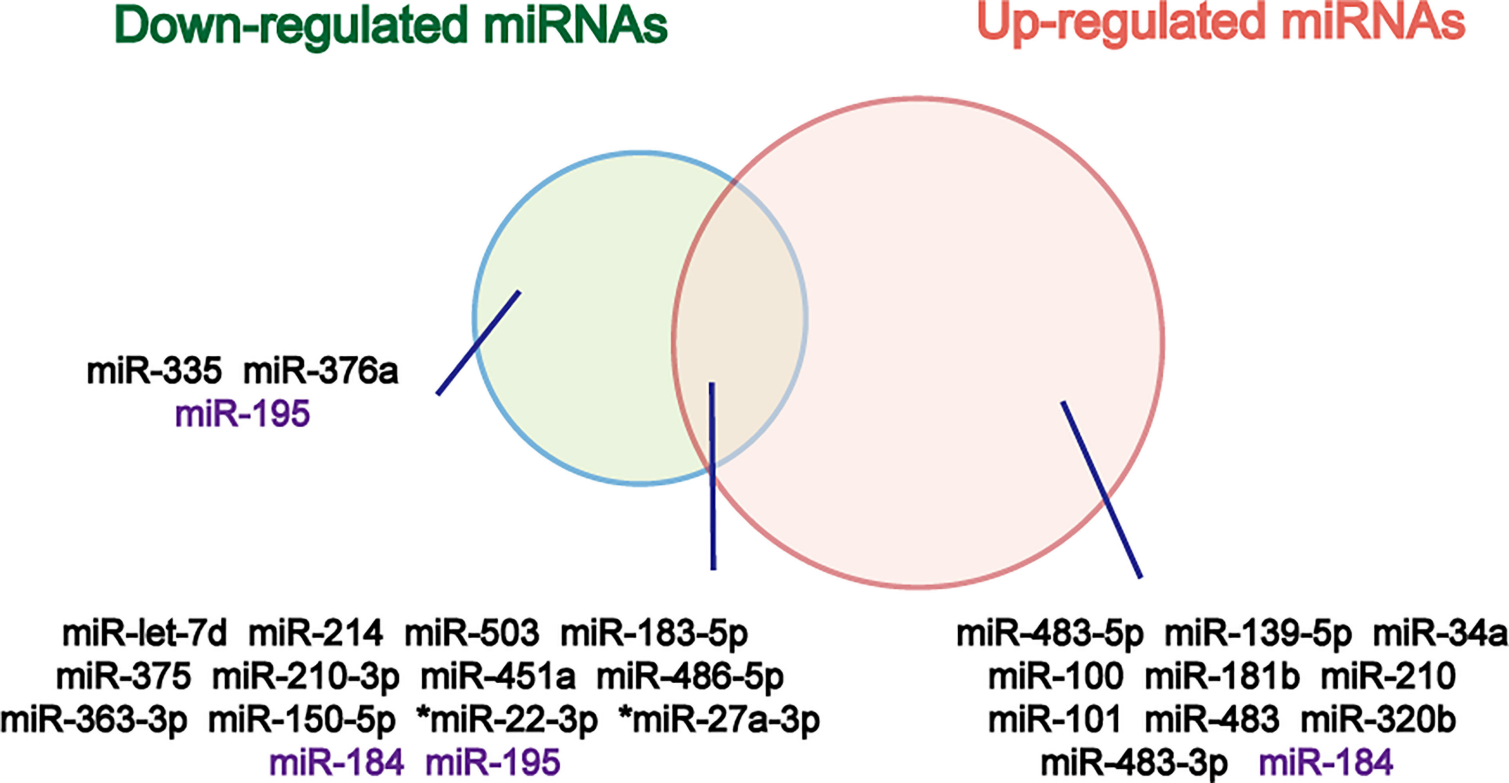
Venn diagram of down- and up-regulated miRNAs in ACC relative to ACA. The miRNAs that don’t show same results in different studies are marked purple. *Overexpressed in both cortisol-producing ACC and cortisol-producing ACA.

Circulating counterparts of these miRNAs in ACC have been detected in several studies, as well **(** **Table 1** **)**. By analyzing the results of these studies, a great number of deregulated miRNAs were identified and validated (**Figure 2**). Deregulated plasma or serum miRNAs have been found by comparing miRNA absolute levels or using dCT method (△CT value equals target miRNA’s CT minus internal control miRNA’s CT) between ACC and ACA (24, 36, 39–44).

**Table 1:** Findings on circulating miRNAs in adrenocortical cancer.

Author and year of publication	Sample type	Method of miRNA isolation	Cohort	miRNAs in ACC compared to adenoma or normal adrenal cortices	Reference
Chabre et al. (2013)	Serum	qRT-PCR	23ACC, 14ACA, 19NA	miR-483-5p↑, miR-335↓, miR-195↓, miR-139-5p↑, miR-376a↓	(24)
Patel et al. (2013)	Serum	qRT-PCR	17ACC, 22ACA	miR-483-5p↑, miR-34a↑	(39)
Szabo et al. (2014)	Plasma	qRT-PCR	13ACC, 12ACA	miR-100↑, miR-181b↑, miR-184↑, miR-210↑, miR-483-5p↑	(40)
Perge et al. (2017)	Plasma-EV	qRT-PCR	22ACC, 24ACA	miR-101↑, miR-483-5p↑	(41)
Salvianti et al. (2017)	Plasma	qRT-PCR	27ACC, 13ACA, 10NA	miR-483-5p↑	(42)
Perge et al. (2018)	Plasma-EV	qRT-PCR	9CP-ACC, 13CPA, 13NFA	miR-320b↑, miR-27a-3p↑, miR-22-3p↑, miR-210-3p↑	(43)
Decmann et al. (2018)	Plasma	qRT-PCR	11ACC, 11ACA, 11AML	miR-483-3p↑, miR-483-5p↑	(36)
Decmann et al. (2019)	Plasma	qRT-PCR	23ACC, 23ACA	miR-483-5p↑	(44)

↑, up-regulation; ↓, down-regulation; qRT-PCR, quantitative real-time polymerase chain reaction; ACC, adrenocortical carcinoma; ACA, adrenocortical adenoma; AML, adrenal myelolipoma; NA, normal adrenal; EV, extracellular vesicle; CP-ACC, cortisol-producing adrenocortical carcinoma; CPA, adrenocortical adenoma; NFA, non-functioning adrenocortical adenoma.

Overexpressed plasma or serum miR-483-5p has been found in nearly all studies, and the expression of circulating miR-483-5p was not influenced by hormonal tests used in routine diagnosis of cortisol overproduction (22). Based on the findings above, circulating miR-483-5p can be served as the best biomarker for diagnosis of ACC. However, in Chabre et al. study, ACCs were subdivided into patients with aggressive ACC (aACC) and patients with no-aggressive ACC (naACC). Serum miR-483-5p was detected neither in healthy people nor in ACA or naACA patients. On the contrary, serum from patients with aACC displayed a high level of miR-483-5p (median value: 472 000 copies/ml) (24). In another study, circulating miRNAs were evaluated in patients with ACC, ACA and adrenal myelolipoma (AML), and no significant difference of miR-483-5p expression was found in ACC and AML samples (36). Results of these two studies might limit the clinical utility of circulating miR-483-5p for diagnosis of malignancy.

Salvianti et al. have measured miR483 and miR-483-5p absolute levels in plasm samples, and significant overexpressions of miR-483 and miR-483-5p were detected in advanced stages of ACC (stage III-IV) relative to early stages (stage I-II), ACA and normal adrenal cortices (42). A correlation between miR-483-5p levels and the number of circulating tumor cells has also been discovered (42). In another study, a correlation between urinary and plasma expression of miR-483-5p has been found (44). However, no significant difference of expression of miR-483-5p has been identified in urine samples between ACC and ACA (44).

There is no recommendation whether plasma or serum is more suitable for evaluation of extracellular miRNAs. A study observed RNAs in serum samples and in the corresponding plasma samples, the higher RNA concentration in serum samples have been found. The reason for this phenomenon was that addition RNA was released from cell during the coagulation process, which demonstrated the miRNAs in serum could be affected by this process (45). Using plasma to extract miRNAs was not affected by above process, but cellular contamination such as platelets can occur easily (46). Several studies have investigated the expression of EV-associated miRNAs, which are secreted actively and could be more sensitive and specific (41). Perge et al. have investigated the expression of EV-associated miRNAs and their applicability for diagnosis of ACC. Overexpressed miR-483-5p and miR-101 have been found in ACC compared to ACA in this study (41).

As for reference genes, synthetic spike-in-RNAs (e.g. cel-miR-39) has been used as reference in several studies (24, 40, 41), and Circulating miRNAs with relatively stable expression like miR-16 was useful in some studies, as well (20, 39, 40). In previous studies, researchers have found that miR-16 and cel-miR-39 were applicable for normalization. However, it is still a debate which is the most optimum method for miRNA normalization in qRT-PCR analysis.

The diagnostic accuracy of these miRNAs was different **(** **Table 2** **).** Extracellular vesicle (EV) associated miR-483-5p appeared to be the most promising minimally invasive biomarker of ACC with the highest accuracy (41). Meanwhile, miR-195 in serum had a high diagnostic accuracy as well (24). However, the sensitivity and specificity reported for these circulating miRNAs are not high enough for clinical diagnosis of malignancy at present.

**Table 2:** Diagnostic accuracy of circulating miRNA for differentiating adrenocortical carcinoma from adrenocortical adenoma.

Author and year of publication	Tape of sample	Comparison	MiRNA	Sensitivity	Specificity	AUC	Reference
Chabre et al. (2013)	Serum	ACC-ACA	miR-195	90.9	100	0.948	(24)
			miR-335	95.2	71.4	0.837	
			miR-139-5p	87.5	65	0.714	
			miR-376a	71.4	85.7	0.811	
		aACC-naACC	miR-483-5p	87.5	100	0.929	
Patel et al. (2013)	Serum	ACC-ACA	miR-34a	ND	ND	0.81	(39)
			miR-483-5p	ND	ND	0.74	
Szabo et al. (2014)	Plasm	ACC-ACA	dCT_miR-210_-dCT_181b_	88.9	75	0.87	(40)
			dCT_miR-100_-dCT_181b_	77.8	100	0.85	
Perge et al. (2017)	P-EV	ACC-ACA	miR-483-5p	87.5	94.44	0.965	(41)
			miR-101	68.75	83.33	0.766	
Salvianti et al. (2017)	Plasma	Low stages ACC-High stages ACC	miR-483	87.5	63.6	0.875	(42)
			miR-483-5p	83.3	100	0.917	
Perge et al. (2018)	P-EV	CP-ACC-CPA	miR-320b	88.89	76.92	0.8632	(43)
Decmann et al. (2018)	Plasma	ACC-ACA	miR-483-5p	81.82	90.91	0.88	(36)
Decmann et al. (2019)	Plasma	ACC-ACA	miR-483-5p	87	78.3	0.88	(44)

miR, microRNAs; P-EV, Plasma-extracellular vesicle; AUC, area under curve; ACC, adrenocortical carcinoma; aACC, aggressive ACC; naACC, non-aggressive ACC; CP-ACC, cortisol-producing ACC; CPA, cortisol-producing ACA; Tumor stage was assessed according to the revised TNM classification of ACC, low stages(stage I-II); high stages(stage III-IV). ND, no data.

The expression of some circulating miRNA might be associated with cortisol secretion. Perge et al. evaluated the expression of plasma extracellular vesicle (EV)-associated miRNAs in patients with non-functioning adrenocortical carcinoma (NFA), cortisol-producing adrenocortical adenoma (CPA) and cortisol-producing adrenocortical carcinoma(CP-ACC). The expressions of miR-22-3p, miR-27a-3p, and miR-320b were significantly increased in both CP-ACC and CPA compared to NFA. The expression of miR-320b was detected to be overexpressed in CP-ACC relative to CPA (43). Igaz et al. have investigated the effect of adrenocorticotropin and dexamethasone on the expression of miRNAs. MiR-27a expression can be regulated by hormone both *in vitro* and *in vivo (* 47). Based on these findings, it is supposed that cortisol overproduction might play a role in expressions of some miRNAs.

The role of miRNAs as prognostic markers in patients of ACC have been investigated in several studies. Chabre et al. has reported for the first time that shorter overall survival was associated with increased miR-483-5p and decreased miR-195 expression in circulation (24). A recent study, measuring miR-483-5p levels three months after surgery, has found that serum miR-483-5p levels were higher in patients with poor prognosis (recurrence or death within 3 years after surgery) than patients with good prognosis (no recurrence for at least 3 years) (48).

Moreover, circulating miRNAs could be applied as non-invasive biomarkers for evaluating treatment efficacy. This application of miRNAs has been investigated in two xenograft studies. The expression of circulating miR-483-5p was significantly reduced by effective mitotane and 9-cis retinoic acid treatment in a mouse NCI-H295R xenograft model (49). MiR-210 ratio in an SW-13 xenograft model was altered after liposomal etoposide-doxorubicin-platina-mitotane (LEDP-M) chemotherapy treatment, which indicated that plasm miR-210 might be a promising biomarker for monitoring treatment efficiency (50).

### Challenges and Perspectives

It must be noted that despite these promising results, applying circulating miRNAs for diagnosis, prognosis, and follow-up is still difficult. It is related to different platforms, different analytical methodologies, lack of standard genes, and the low concentration of miRNAs. Meanwhile, miRNAs are not specific for ACC, which means the same variation could be found in another disease. Furthermore, the number of the patients involved in previous studies was small. In the future, further studies with large cohorts, standard methodologies, and recommended reference genes are warranted.

## Steroid Metabolite Profiling

### Steroidogenesis in Adrenocortical Carcinoma

**Figure 3:**
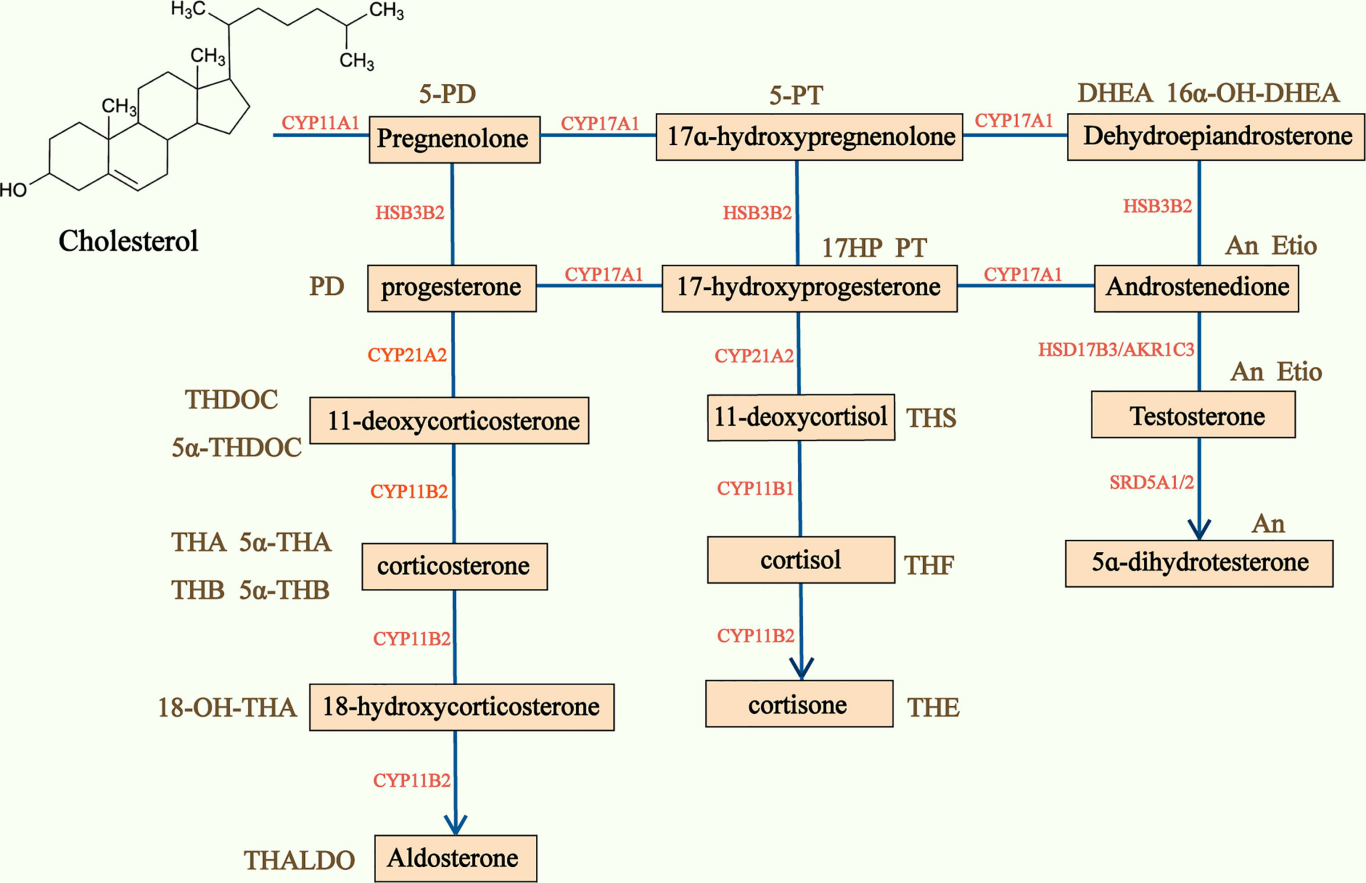
Adrenal steroidogenesis and main corresponding urine steroid metabolites. Steroidogenesis pathway is represented in the center of the figure and the main urine steroid metabolites on the sides of the picture. CYP, Cytochrome P450; HSD, hydroxysteroid dehydrogenase; 5-PD, pregnenediol; PD, pregnandiol; THDOC, tetrahydrodeoxycorticosterone; THA, tetrahydro-11-dehydrocorticosterone; THB, tetrahydrocorticosterone; THALDO, tetrahydroaldosterone; 5-PT, pregenetriol; PT, pregnanetriol; THS, tetrahydro-11-deoxycortisol; THF, tetrahydrocortisol; Et, etiocholanolone; THE, tetrahydrocortisone; DHEA, dehydroepiandrosterone; An, androsterone; Etio, Etiocholanolone.

50-70% of ACC are found to produce adrenal hormones in excess, though this is not clinically apparent in many cases (4, 7). Serum and urinary steroid analysis generally play an important part in diagnosis of adrenal hormone excess and disorders of steroidogenesis. Based on traditional serum steroid analysis, which mostly assesses end products of steroidogenesis, a large number of patients with ACCs are defined as nonfunctioning. However, the steroidogenic pattern produced by dedifferentiated and immature malignant ACC cells is characterized by many steroid precursors and metabolites rather than end products of complete steroidogenesis (51), and precursors and metabolites are often accumulated in patients with ACC (52) (**Figure 3**). Researchers have investigated steroid profiling and have found that steroid profiling has great potential in diagnosis, prognosis and monitoring recurrence in ACC.

### Urine Steroid Profiling

Several decades ago, Grondal et al. have shown that the urine metabolomics might be reliable to identify ACC with high sensitivity and specificity and to predict their recurrence during follow-up (53). More recently, several retrospective studies have described patients with adrenal tumors showing significant differences of steroid excretions between ACCs and ACAs **(** **Table 3** **)**, which suggests significant value of urine steroid metabolite profiling for a highly accurate diagnostic test of adrenocortical tumors.

**Table 3:** Studies on urine steroid profiling for diagnosis of ACC.

Author, year	Samples	Type of study	USP	Methods	Findings	Reference
Gröndal et al. (1990)	24ACC, 10 CS, 8PA, 16control	prospective	Not known	GC-MS	3β-hydroxy-5-ene steroids and/or metabolites of cortisol precursors, such as THS were increased in ACC	(53)
Alrt et al. (2011)	45ACC, 102ACA	Retrospective	32 metabolites	GC-MS	Increased nine steroids in ACC (5-PD, 5-PT, DHEA, 16ɑ-OH-DHEA, THDOC, 5ɑ-THDOC, PD, PT, THS)	(54)
					THS, 5-PT, and 5-PD were most informative steroids in discriminating ACC from ACA	
Kerkhofs et al. (2015)	27ACC, 107ACA, 18 other adrenal conditions	Retrospective	22 metabolites	GC-MS	THS was the most informative maker and excretion of THS was associated with ACC tumor size and stage.	(55)
					THS at a cut-off value of 2.35µmol/24 h differentiated ACC from other adrenal disorders with 100% sensitivity and 99% specificity	
					No significant differences in metabolite excretion between functioning and non-functioning ACC	
Velikanava et al. (2016)	31 ACC, 108 ACA	Retrospective	66 metabolites	LC-MS and GC- MS	100% sensitivity and specificity of ACC and ACA differential diagnosis can be achieved by combining several parameters of urine steroids.	(56)
					Increased THS in 74.2% of patients with ACC	
					Increased DHEA and its metabolites in 67.7% of patients with ACC	
Hines et al. (2017)	114 control, 71 adrenal disease	Retrospective	26 metabolites	HRAM LC-MS	11 steroids with increased Z score, especially tetrahydro-11-deoxycortisol, pregnanetriol, and 5-pregnenetriol	(57)
Shafigullina et al. (2018)	26 control, 103AI	Retrospective	66 metabolites	GC-MS	Increased THS, androgens and progestenes in ACC	(58)
Bancos et al. (2020)	98ACC, 1919 non-ACC mass	Prospective	15 Metabolites	LC-MS/MS	USP indicating high risk of ACC were seen in 83 (84.7%) of 98 ACC and 157 (8.2%) of 1919 non-ACC masses	(59)

ACC, adrenocortical carcinoma; CS, Cushing’s syndrome; PA, primary hyperaldosteronism; ACA, adrenocortical adenoma; GC-MS, gas chromatography-mass spectrometry; DHEA, dehydroepiandrosterone; USP, urine steroid profile; 5-PD, pregnanediol; THS, tetrahydro-11-deoxycortisol; HRAM LC-MS, liquid-chromatography; high-resolution; accurate-mass mass spectrometry; LC-MS, liquid-chromatography mass spectrum.

The first large research of steroid profiling was conducted by Arlt et al. with 45 ACC patients and 102 ACA patients (54). Quantifications of 32 distinct adrenal steroids were carried out by gas chromatography-mass spectrometry(GC-MS) in urine samples. The study indicated that the glucocorticoid precursor metabolite tetrahydro-11-deoxycortisol(THS) and androgen precursor metabolites pregnanediol and pregnanetriol were the most informative steroids in discriminating ACC from ACA. Nine most discriminative steroids for differentiating ACC from ACA have been identified by using the machine learning-based algorithm called generalized matrix learning vector quantization (GMLVQ). Receiver-operating characteristics (ROC) analysis demonstrated that the diagnostic information of 32 steroids resulted in 90% sensitivity and specificity, while the nine most discriminative and the top three steroids provided only slightly lower diagnostic accuracies (87.7% and 87.2%, respectively) (54). Receiver-operating characteristics revealed great performance of GMLVQ.

In Kerkhofs et al. study, they performed GC-MS analysis but did not apply computational analysis of 22 steroid metabolites in 27 ACCs, 107 ACAs, and 8 other adrenal conditions. They found 15 steroid markers with a sensitivity of at least 90% in diagnosing ACCs, but specificities varied from 2% to 83% (55). They showed that THS was the most informative markers again and its excretion was significantly associated with ACC tumor size and stage. In addition, THS at a cut-off value of 2.35umol/24h can detect ACC with a 100% sensitivity and 99% specificity (55).

Velikanova et al. conducted a research to analyze urine steroid profiling by using high-performance liquid chromatography(HPLC) and gas chromatography-mass spectrometry(GC-MS) methods in 31 ACCs and 108 ACAs. By combining several parameters of urine steroids, including THS, pregnanediol, 5-pregnanetriol, and 5-pregnanediol, 100% sensitivity and specificity of ACC, and ACA differential diagnosis can be achieved (56). THS was also confirmed to be the most informative markers. However, increased urinary excretion of THS was only in 74% of ACC patients, and DHEA and its metabolites in 67.7% patients with ACC (56). Hines et al. have developed a novel multiple-steroid assay to quantify 26 urine steroid metabolites by using HRAM LC-MS, and have shown that patients with ACC had 11 steroid metabolites with increased Z scores, especially tetrahydro-11-deoxycortisol (14 vs 0.5, P<0.001), pregnanetriol (7.5 vs -0.4, P=0.001), and 5-pregnenetriol (5.4 vs -0.4, P=0.01), which might improve diagnosis for patients with ACC (57).

These studies found that tetrahydro-11-deoxycortisol (THS) was the most informative steroid marker to discriminate ACC from ACA (54–58). THS is the metabolite of 11-deoxycortisol, which is converted to cortisol by 11β-hydroxylase. The mechanism for THS overproduction in ACC remains unclear, but it seems to be related with deficiency of 11β-hydroxylase and or dysfunction of the enzyme itself. The mechanism of accumulation of other precursors may be similar with THS (59).

We can find that in all the studies above, the excretion of steroid precursor metabolites in ACC is higher than metabolites in ACA. Urine steroid profiling is a promising prognostic method to distinguish ACCs from ACAs with high sensitivity and specificity. However, above studies were based on retrospective studies with small numbers of patients. In order to use urine steroid profiling for distinguishing malignancy from benign, a large prospective study is needed.

Recently, to validate the use of urine steroid metabolomic testing, a multicenter Evaluation of Urine Steroid metabolomics in the Differential Diagnosis of Adrenocortical Tumor(EURINE-ACT) study was conducted. Bancos et al. have validated the diagnostic utility of urine steroid metabolomics in detecting ACCs (59). Previous studies of urine steroid metabolomics for ACC detection analyzed urinary steroid profiling by gas chromatography-mass spectrometry, which is a low-throughput method requiring highly specialized expertise. In this study, Bancos et al. used the high-throughput LC-MS/MS approach, which is more widely available in clinical routine. The accuracy of diagnosis of urine metabolomics was higher than maximum diameter and imaging characteristics in this study. When combining these three parameters, the positive predictive value was 76.4% (59).

Urine steroid metabolites can not only hold promise for early diagnosis but also provide a non-invasive biomarker for the follow-up of patients with ACC. Several studies have shown that the detection of steroid metabolites in urine can predict recurrence before imaging evidence is found; and can be used to assess completeness of resection after surgery (60, 61). Early detection of recurrence may allow timely cytotoxic chemotherapy and radical surgery, which can improve the overall survival. It is noted that the levels of urine steroid are associated with the bulk of malignant lesions. Mitotane is the most widely used chemotherapy medications in adrenocortical carcinoma, which interferes with steroidogenesis in various ways. Nevertheless, Chortis et al. have found that the diagnostic performance of the steroid profile was not influenced by adjuvant mitotane treatment (60).

### Plasma Steroid Profiling

Steroid profile in urine samples allows a non-invasive integrated assessment of daily steroid production, which provides a high sensitivity and specificity in differentiating ACC from non-ACC lesions (62). However, the analysis of steroid profile in urine still has many limitation. GC-MS poor practicability and the inaccuracy of 24-h urine collection restrict to transfer this technique into clinical practice (63).

Plasma samples are easier to collect than 24h urine samples. Two studies have examined steroid profiling by using LC-MS/MS in serum to investigate their value in diagnosis and monitoring recurrence of ACC. Liquid-chromatography mass spectrum were used in both studies, which is readily accessible in clinical routine (64). In a very small study with only ten ACC patients, Taylor et al. have developed an LC-MS/MS method for measurement of a 13-steroid panel. 4-7 steroids (median=6) were increased in ACC cases, while 11-Deoxycortisol was markedly increased in all cases of ACC. A complete separation between ACC and non-ACC group could be achieved by using the whole 13-steroid panel. 11-deoxycorticol and 17-hydroxypregnenolone were the best markers to discriminate ACC from non-ACC (65).

More recently, Schweizer et al. have quantified 15 steroid hormones in 66 ACA and 42 ACC plasma samples by combining multiple statistics into a machine-learning model. Higher abundances of 11-deoxycorticosterone, progesterone, 17-hydroxyprogesterone, 11-deoxycortisol, DHEA, DHEAS and estradiol have been observed in ACC (66). In comparison with the study of Arlt’s group, the AUC values of their model (six steroid hormones) are significantly smaller than those obtained with nine steroid metabolites in 24h urine. The model could not be improved by inclusion of all measured plasma steroids (66). It may be that the large proportion of tumors in Arlt et al. study were in advanced stages, which may be associated with more prominent alterations of steroid hormones and precursors. Meanwhile, in Alrt et al. study, a larger number of ACAs were included (54). A conclusion regarding the optimal matrix and method of analysis cannot be drawn yet.

### Challenges and Perspectives

Based on what’s been discussed above, the diagnostic and prognostic value of steroid profiling in ACC has been confirmed. However, there are still many challenges. The majority of measurements of steroid profiling are rely on GC-MS. The GC-MS can only be manufactured by a small number of laboratories and institutions. Meanwhile, a lack of harmonization between laboratories has limited its further investigations and clinical applications of it. Detection of a steroid profiling is a time-consuming examination, so it cannot be used for screening of all patients with ACC at present. Therefore, developing simple methods for analysis of specific steroid profiling is crucially important. Owing to the low incidence of adrenocortical carcinoma, studies often conclude a few cases. Prior to implementation in routine clinical practice, steroid profiling as a diagnostic tool need to be validated in a larger cohort.

## Circulating Tumor Cells

Another prospective area of ACC is the study of circulating tumor cells(CTCs). Circulating tumor cells (CTCs) are neoplastic cells shed into the blood stream from either primary tumor or metastases (67–70). They can be differentiated from patients’ surrounding normal hematopoietic cells. Approaches to CTCs isolation include a large panel of technologies, which were based on different properties (physical and biological properties) of CTCs. Physical properties of CTCs include density, size, electric charges, deformability and so on, while biological properties include cell surface protein expression and viability (71). The advantage of using physical properties is that they allow CTC separation without labeling (71). Detection of circulating CTCs in peripheral blood is a useful tool for diagnosis, prognosis, and follow-up in several solid cancers, especially breast cancer (67, 68, 72–74).

A preliminary study has revealed that CTCs are present in circulation of patients with ACC but not in patients with ACA, providing the first evidence that CTCs might be a valid and useful presurgical marker to differentiate between malignant and benign adrenocortical tumors (67). Meanwhile, a significant decrease in the number of CTCs has been found after surgery compared to presurgical samples, which illustrates that CTCs can be a promising biomarker for follow-up (67).

The results of the recently published research have shown that CTCs (obtained from liquid blood biopsy) were presents in 68% of pre-surgery and in 38% of post-surgery blood samples, which illustrates that CTCs can be a promising marker for monitoring disease in ACC (75). Patients were stratified in high and low pre-surgery CTC number groups (75th percentile CTC value as cut-off), and CTCs significantly predicted the patients’ overall survival (75). Meanwhile, CTCs provide a minimal invasive method that can be used to study genetic information, which will contribute to our understanding of adrenocortical tumors (76). Both studies above isolated CTCs from blood by ScreenCell devices system (ScreenCell), which were based on cell size and morphological criteria. Immunocytochemistry was performed on enriched CTCs and their origins of ACC were confirmed. Although with the steady development of techniques, CTC isolation has become more feasible over the past years, CTC identification and characterization remain challenging.

These studies provide a promising utility of CTCs for diagnosis and follow-up of ACC. However, they were both based on small cohorts of patients. Further studies with larger cohorts of patients are needed to confirm the diagnostic and prognostic value of CTCs in adrenocortical carcinoma before we can establish its clinical utility.

## Circulating Cell-Free Tumor DNA

Circulating cell-free DNAs (cfDNAs) are short extracellular DNA fragments (approximately 160-180bp) in body fluids that are derived from the remnants of “healthy cells”, malignant cells, and tumor microenvironmental cells (77, 78). Circulating cell-free DNA is predominantly resulted from enzymatic degradation during or after cell death (79). In patients with ACC, a variable fraction of cfDNA in body fluids is contributed by cancer cells. These cfDNAs, known as circulating tumor DNA(ctDNA), carrying tumor-specific somatic genetic alterations, can be released by the primary tumor, metastasis, and circulating tumor cells. The amount of ctDNA is largely depends on the tumor type, diameter and disease stage (80). The mechanism by which the tumor sheds DNA into the blood is still obscure (81). Detection of somatic mutations that are specific to cancer cells helps discriminate circulating cell-free tumor DNA(ctDNA) from ccfDNA of non-tumoral origin (82).

The poor quality of ctDNA and its extremely low levels makes qualification and detection quite difficult (79). The development of highly sensitive technologies, such as digital PCR and Next Generation Sequencing enables researchers to obtain ctDNA profiling and compare it with the profiling of the primary lesions to gain deep insight into the heterogeneity and clonal evolution of the tumor (77).

The role of ctDNA as a biomarker for diagnostic, prognostic, and therapeutic monitoring, has already been investigated in many types of cancers (82, 83). Recently, ctDNA has been investigated in ACC as well. There are two studies that have detected the mutation in cfDNA within serum samples of patients with ACC.

Creemers et al. performed a pilot study including six patients with ACC. Mutations have been found in primary tumors in three of them by Next-generation sequence (NGS). Cell-free circulating DNA (cfDNA) was isolated from blood samples of these patients, which were collected before (1 to 2 weeks) and after surgery. Tumor-specific mutations were only found in one of these three patients who had metastatic ACC at diagnosis (84). Preoperative cfDNA showed the same mutations as primary tumor by NGS, while postoperative cfDNA showed the same mutations but at lower frequencies. In the cfDNA from two patients with known mutations in primary tumor, tumor-specific mutations can’t be detected, which suggests that this minimally invasive approach will only be suitable for detecting disease progression in a subgroup of patients with ACC (84). They detect mutations in primary tumor by NGS to identify these tumor-specific mutations in cfDNA. The limitation of this method is that the tumor-specific mutations, which were detected in cfDNA, must be present in primary tumor and can be detected by NGS (84).

In another study, ctDNA was detected by highly sensitive technologies (deep NGS and droplet digital RCR). Garinet et al. also found that ctDNA only presented in a subset of ACC patients. In this study, eight patients were detected of at least one mutation in the tumor, while mutations in the cfDNA were only found in two out of these eight patients. Though two of the currently most sensitive molecular biology technologies were used, it remains challenging to conclude whether ctDNA detection was negative because of a limited sensitivity (85).

These two results indicate that different ACC patients were associated with different levels of ctDNA. Compared with CTCs, ctDNA is much easier to isolate and more sensitive to detect. However, application of ctDNA as a potential biomarker for ACC remains a problem. Further studies with larger cohorts, longitudinal monitoring, and standardized methodologies are needed in the future.

## Radiomics

The main challenge in the management of adrenal incidentalomas is to differentiate malignant from benign lesions. CT is the dominant imaging tool for evaluating the adrenocortical tumors. Size is thought to be the most important predictor for malignancy, and a size of > 4cm is a crucial feature (86). A large retrospective single-center study has found that 70% larger adrenal tumors (> 4cm) turn out to be non-malignant lesions (87). Therefore, this risk factor (diameter) should not be taken in isolation for diagnosis of malignancy. Machine learning has been used to differentiate large (> 4cm) adrenocortical carcinoma from other large adrenocortical lesions on contrasted-enhanced CT. The radiomics, obtained by machine learning had a diagnostic accuracy (82%) for malignant tumor exceeding that of radiologists (68.5%) (88). Meanwhile, Torresan et al. have also found that CT texture analysis by an unsupervised machine learning approach could predict malignancy in nearly all patients (89). These two studies have illustrated that radiomics could be served as a novel non-invasive tool to differentiate benign from malignant adrenocortical tumors. For clinical perspective, other non-invasive biomarkers (miRNA, steroid profiling and so on) requires a long time to collect and analyze data. Meanwhile these biomarkers are not available in all countries. On the contrary, computed tomography is used in all clinics and only a dedicated software is required. However, these two studies have limitations related to their design and relatively small sample size. Further studies on larger patient populations are warranted.

## Conclusion

In spite of all the progress that has been made, it seems that we are far from utilizing non-invasive biomarkers to diagnose a malignant adrenocortical tumor and predict prognosis. Although many problems remain unsolved, future research directions of early diagnosis and detection of recurrence of ACC may still revolve around these non-invasive biomarkers. We expect that non-invasive biomarkers will be used in clinical practice in the future and will improve the management of adrenocortical carcinoma.

## Author Contributions

YZ designed and revised the manuscript. YC, WK, DZ, and XY wrote the manuscript and made the figures. All authors contributed to the article and approved the submitted version.

## Funding

This work was supported by the grants from the National Science Foundation of Shanghai (NO.21ZR1440700).

## Conflict of Interest

The authors declare that the research was conducted in the absence of any commercial or financial relationships that could be construed as a potential conflict of interest.

## Publisher’s Note

All claims expressed in this article are solely those of the authors and do not necessarily represent those of their affiliated organizations, or those of the publisher, the editors and the reviewers. Any product that may be evaluated in this article, or claim that may be made by its manufacturer, is not guaranteed or endorsed by the publisher.
